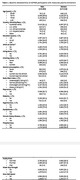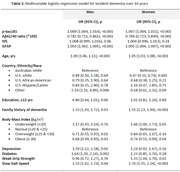# Sex‐specific associations between plasma biomarkers and risk factors for dementia: a 10‐year follow‐up of community‐dwelling older individuals

**DOI:** 10.1002/alz.095449

**Published:** 2025-01-09

**Authors:** Joanne Ryan, Anne Murray, Zimu Wu, Michelle M. Mielke

**Affiliations:** ^1^ Monash University, Melbourne, VIC Australia; ^2^ Berman Center for Outcomes and Clinical Research, Hennepin Healthcare Research Institute, Minneapolis, MN USA; ^3^ University of Minnesota, Minneapolis, MN USA; ^4^ Wake Forest University School of Medicine, Winston‐Salem, NC USA

## Abstract

**Background:**

Evidence is growing supporting sex‐ and gender‐ specific differences in risk factors for dementia, and varying pathways to diagnosis. Recognized sex differences in brain structure and function could contribute to differences in plasma biomarker concentrations and in susceptibility to brain pathologies. Furthermore, differences in comorbidities between males and females (e.g. kidney function) could influence sex differences in the clearance and measurement of these biomarkers. The aim of this study was to investigate the association between risk factors and plasma biomarkers, with incident dementia, separately in men and women.

**Method:**

In the U.S and Australia, we recruited 19,114 community‐dwelling individuals aged ≥70 years (U.S African‐Americans and Hispanics ≥65 years), without cognitive impairment and cardiovascular disease. Plasma amyloid‐beta (Aβ40 and Aβ42), phosphorylated tau (p‐tau 181), neurofilament light (NfL), and Glial Fibrillary Acidic Protein (GFAP) were measured [Simoa N4PE and Ptau181 v2 assays] at baseline. Dementia was adjudicated by an expert panel according to DSM‐IV criteria. The association between a range of factors (Table 1), and the risk of dementia was assessed using multivariable logistic regression, separately in men and women.

**Result:**

Participants had a mean age of 74 years at baseline and plasma biomarker data was available for 7,218 women and 5,942 men. Of these participants, 824 developed dementia, with a similar proportion of men (6.4%) and women (6.2%). Age, a family history of dementia, BMI and slow gait speed were significantly associated with dementia risk, in both men and women (Table 2). All of the plasma biomarkers were associated with dementia risk, but NfL was not significant after adjustment for GFAP (particularly in women). In men only, depression and diabetes were significant risk factors, while in women poor grip strength was associated with increased risk, and U.S whites compared to Australian whites, had decreased risk.

**Conclusion:**

Plasma biomarkers of Alzheimer’s Disease were found to be associated with dementia risk in both men and women, however associations between other risk factors and dementia varied. These findings suggest the importance of considering sex and gender differences, as this could open up possibilities for personalized prevention.